# One-Pot Growth of 2D/3D Hybrid Perovskite Vertical Heterostructures

**DOI:** 10.1021/acsmaterialslett.5c01109

**Published:** 2025-11-06

**Authors:** Selene Matta, Valeria Demontis, Angelica Simbula, Riccardo Pau, Simone Argiolas, Alessandro Mattoni, Silvia Liscia, Ruirui Wu, Nan Zhao, Aditya Bhardwaj, Francesco Mattana, Emanuele Cadeddu, Prajkta Liladhar Nehete, Nicola Sestu, Daniela Marongiu, Francesco Quochi, Michele Saba, Andrea Mura, Giovanni Bongiovanni

**Affiliations:** ‡ Dipartimento di Fisica, Università degli Studi di Cagliari, 325871 Cittadella Universitaria, 09042 Monserrato (CA), Italy; § Dipartimento di Fisica, Università degli Studi di Cagliari and CNR - Istituto Officina dei Mateiali (IOM) Cagliari, Cittadella Universitaria, 09042 Monserrato (CA), Italy; ∥ CNR − Istituto Officina dei Materiali (IOM) Cagliari, Cittadella Universitaria, 09042 Monserrato (CA), Italy

## Abstract

Single-crystal metal-halide perovskites hold significant promise for optoelectronic applications due to their tunable physical properties and the possibility of low-cost, low-temperature synthesis. Compared to their polycrystalline counterparts, they exhibit reduced defect densities and enhanced stability. Their intrinsically soft lattice facilitates integration with conventional semiconductors via heterostructures. However, their high ionic mobility can lead to interdiffusion processes that compromise the integrity of adjacent layers, making the formation of well-defined interfaces a critical challenge for device optimization. Here, we exploit the temperature dependence of the perovskite growth kinetics to demonstrate a one-pot, space-confined growth method for synthesizing vertical 2D/3D lead-halide perovskite heterostructures in single-crystal form. The process leverages differences in precursor solubility to drive sequential crystallization and create well-defined interfaces. Structural and optical analyses confirm the formation of stable, phase-separated heterostructures, which are promising for optoelectronic applications.

## Graphical abstract

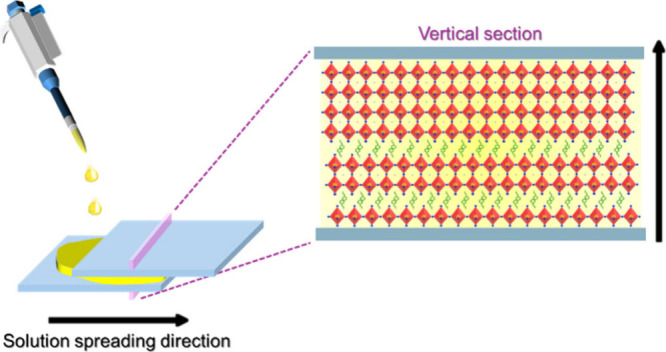

Hybrid perovskites have revolutionized the field of optoelectronics due to their exceptional properties, including high absorption coefficients, tunable bandgaps, and long carrier diffusion lengths.^1–5^ However, device-grade perovskites are typically fabricated as polycrystalline films, which suffer from significant drawbacks such as grain boundaries, structural disorder, and rapid degradation.^6–16^

Single-crystal perovskites overcome many of these limitations by offering superior charge carrier mobility^17–25^ and improved environmental stability, as well as reduced structural defects, providing a more reliable platform for high-performance optoelectronic devices.^17–26^ Among hybrid perovskites, two-dimensional (2D) Ruddlesden–Popper (RP) perovskites offer enhanced environmental stability^27–30^ compared to their three-dimensional (3D) counterparts,^6–10,12^ due to their layered structure and organic–inorganic composition. Conversely, 3D perovskites provide excellent charge transport and light absorption/emission, but are less stable. Single-crystal 2D/3D hybrid perovskites combine the advantages of both phases, stability from 2D layers and efficient charge transport from 3D domains, offering a defect-reduced platform for multifunctional, high-performance devices.^31^

Nevertheless, the growth of thin single-crystal films suitable for device integration remains a significant challenge.^32^ In this work, we present a novel method to fabricate 2D/3D single-crystal heterostructures with controlled composition and thickness, highlighting the potential of reversible growth mechanisms and sharp interface engineering.

Recent developments have shown that compositional grading in mixed-halide bulk single-crystal perovskites can be achieved via continuous-flow synthesis, offering improved control over crystallization dynamics compared to conventional batch processes.^33^ Additionally, efforts have focused on lateral epitaxial heterostructures in 2D halide perovskites enabling the formation of compositionally defined superlattices with reduced interdiffusion and improved interface quality.^34–36^ More recently, vertically stacked low-dimensional perovskite heterostructures obtained by mechanical exfoliation and transfer have been investigated, revealing ultrafast charge transfer at the 2D/3D interface on the picosecond time scale.^37^ While these results provide valuable insight into the interfacial photophysics of low-dimensional systems, the approach is intrinsically limited to micrometer-scale flakes, making device integration challenging. In this work, we present a fundamentally different strategy: the fabrication of thin-film single-crystal perovskite vertical heterostructures combining both 3D and layered (2D) phases within a single monolithic crystal from a single solution bath, thus overcoming the limitations of layer-by-layer growth techniques. The growth results in a vertically stacked, compositionally and structurally engineered multimaterial perovskite film with sharp interfaces and reduced thickness, representing a unique strategy for integrating diverse functional properties in single-crystal devices.

The space-confined growth method is one of the most widely used approaches for synthesizing thin single-crystal perovskite layers with precise thickness control.^38–41^ This technique involves depositing the precursor solution between two substrates, thereby restricting the available space for crystal expansion in the vertical direction. Using this method, the growth of single-crystal vertical heterojunctions with areas in the mm^2^ range and thicknesses below a few micrometers has been successfully demonstrated.^42^ Figure 1 illustrates the growth protocol for synthesizing vertically stacked single-crystal heterostructures. A few drops of the precursor solution are placed between two quartz slides, followed by an extended thermal treatment (see the Supporting Information (SI)). The space-confined approach is not compatible with standard in situ characterization techniques such as XRD or electron microscopy. However, in situ optical inspection using white-light microscopy is a suitable method for real-time monitoring of crystal growth dynamics. To this purpose, we designed a customized growth reactor described in the . Figure 1(c) presents photographs capturing different stages of the process, revealing that the 3D phase initially forms during the heating step up to 140 °C, while the 2D phase starts to emerge during the subsequent cooling phase around 110 °C, after partial solvent evaporation, and continues to develop during the plateau at 100 °C. The proposed growth mechanism is based on the well-known inverse temperature crystallization of 3D hybrid perovskites^43–45^ and the direct solubility of the 2D phase.^46–48^

**Figure 1:**
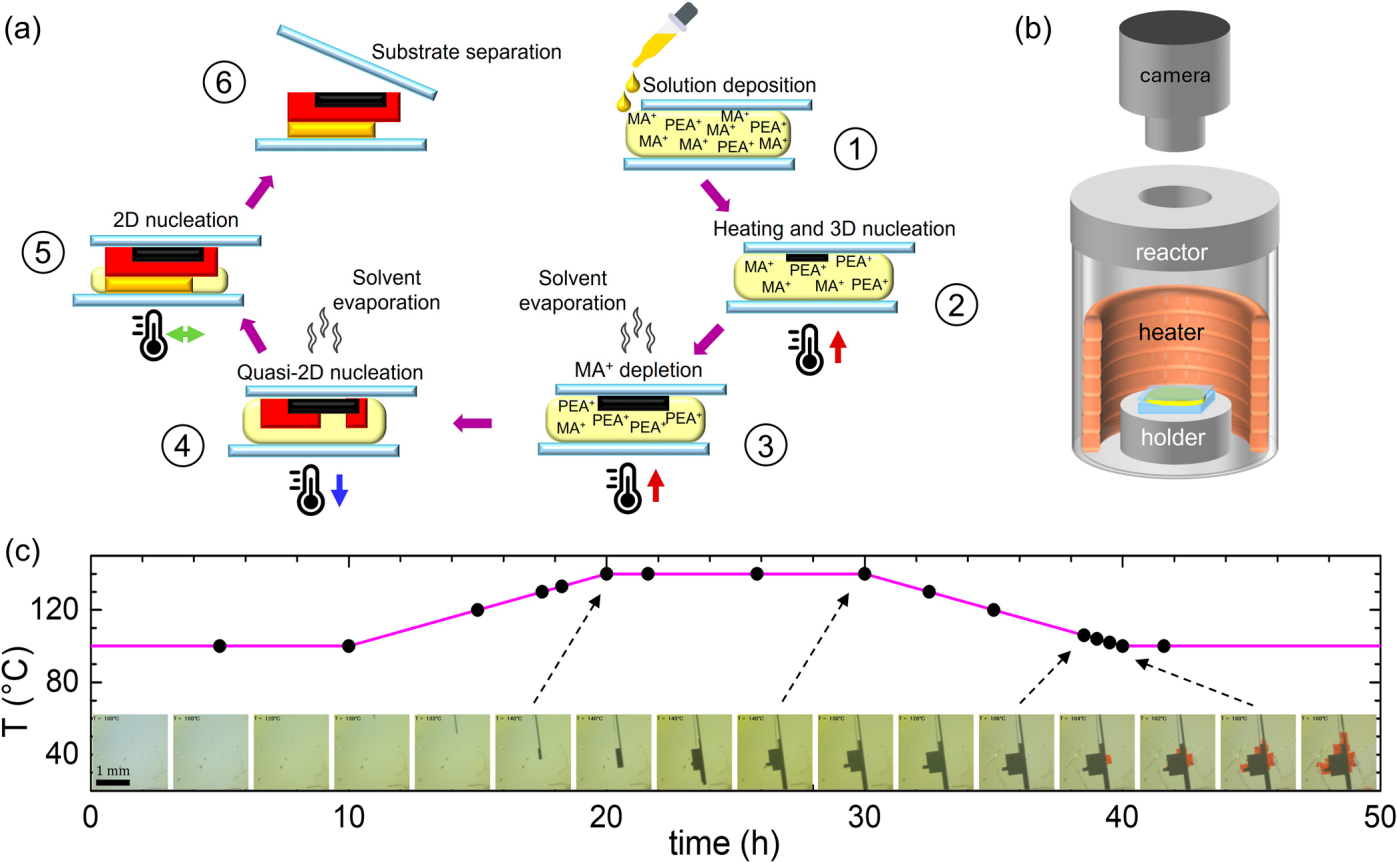
(a) Schematic representation of the fabrication process: (1) solution deposition on a quartz substrate and overlaying with another slide to create a confined space; (2) heating of the system and 3D perovskite nucleation; (3) growth of 3D perovskite crystal with simultaneous partial solvent evaporation and MA^+^ depletion; (4) formation of the quasi-2D perovskite crystal; (5) formation of the additional 2D perovskite crystal; (6) separation of the top slide. (b) Illustration of the heating setup used for in situ observation of the crystallization process: the sample is placed under an optical system and consists of a heating resistor embedded in a PTFE reactor, with a quartz window on top allowing visual access to the sample for real-time monitoring with a camera. (c) Time–temperature protocol showing the temperature variation applied during synthesis. Insets show time-lapse optical microscopy images showing the evolution of crystal growth and phase formation. Images were taken at the corresponding time and temperature black dots, revealing the gradual development of the perovskite heterostructure. Schematic illustration of the crystallization pathway as a function of temperature is also provided, highlighting the sequence of 3D nucleation, subsequent 3D growth with MA^+^ depletion, and the emergence of lower-dimensional 2D phases upon cooling.

Initial crystallization results in the formation of the 3D MAPbI_3_ phase, followed by 2D phases MA_*n*–1_(PEA)_2_Pb_*n*_I_3*n*+1_ (*n* = 2 and *n* = 1). Temperature dependent experiments confirmed the reversible nature of the growth, enabling influence over layer thickness and phase composition. The interplay between the specific growth conditions imposed during the process, and the differences in the temperature-dependent solubility between the two materials, enable the formation of the 3D perovskite in the first part of the temperature ramps (heating phase) and of the 2D perovskites in the second part (cooling phase).

Specifically, during the heating process, the inverse solubility of the solution triggers the nucleation of the 3D phase, leading to the formation of a well-defined squared-shaped single crystal. The 3D crystal growth is limited by the depletion of MA^+^ precursors in the remaining solution. The subsequent nucleation of the 2D phase occurs, as illustrated in Figure 1(c), as the temperature decreases and, at the same time, the concentration of the remaining solution increases due to the partial solvent evaporation from the quartz edges. This process is both reversible and reproducible as demonstrated by the timelapses shown in .

The strength of this method lies in its ability to selectively promote the growth of a specific phase by halting the reaction at a precise stage. Specifically, the formation of the 2D phase can be stopped by controlling the temperature and separating the two quartz slides at the appropriate moment, thereby effectively freezing the system. Moreover, depending on the rate of solvent evaporation, either a single or a double heterostructure can be obtained, allowing additional control over the final architecture and composition of the crystal (see the SI). Full characterization of the samples is carried out using micro X-ray diffraction (μXRD), optical and electron microscopy, and microphotoluminescence. To evaluate the structural integrity and interface quality of the synthesized heterostructures, we performed detailed morphological and structural analysis using scanning electron microscopy (SEM) and scanning transmission electron microscopy (STEM), as illustrated in Figure 2. The region of interest was first identified with an optical microscope, and a cross-sectional lamella was prepared via focused ion beam (FIB) milling (see Figure S2). Figure 2(b) shows the STEM image of the lamella taken in the green area highlighted in Figure 2(a). The heterostructure reveals three well-defined layers: a bottom phase with a thickness of 760 nm, a central layer 3.8 μm thick, and a top layer 800 nm thick. Depth-resolved elemental analysis of the vertical layer stack yielded only an averaged signal rather than the composition of individual layers. This limitation is likely due to the focused ion beam sample preparation, which introduces mechanical and thermal stress, especially critical for soft materials such as hybrid perovskites and organics. Despite optimization to avoid macroscopic damage, surface defects, cross-contamination, and interlayer material transport were unavoidable, making correlation with complementary characterization techniques essential for a comprehensive analysis.

**Figure 2:**
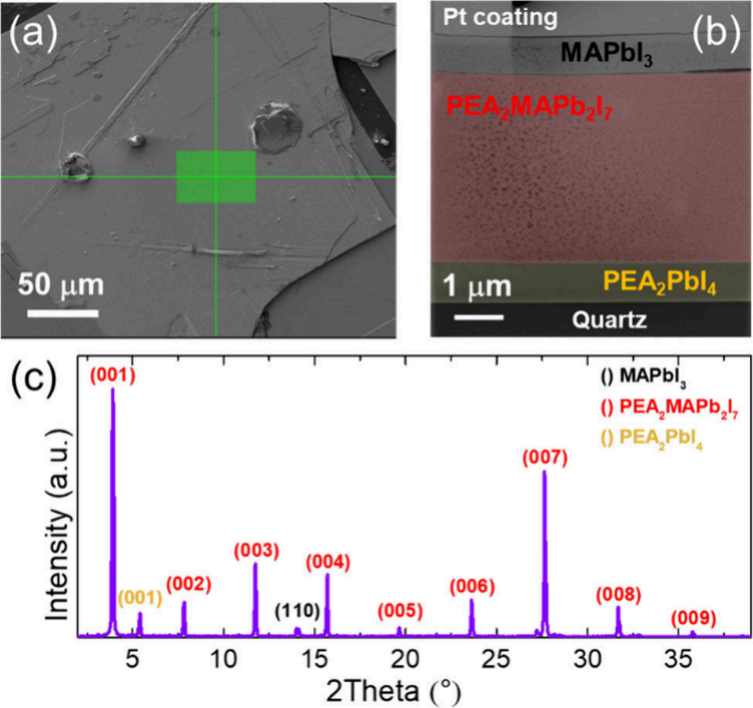
(a) SEM image of the sample surface with the analyzed region highlighted in green. (b) Cross-sectional STEM image of a lamella extracted from the region indicated in panel (a), illustrating a well-defined three-layer heterostructure composed of PEA_2_PbI_4_ (bottom, yellow), PEA_2_MAPb_2_I_7_ (middle, red) and MAPbI_3_ (top, gray). (c) X-ray diffraction pattern collected from the same area prior to lamella preparation, displaying reflections attributed to the three crystalline phases.

The layer composition has been determined by micro-X-ray diffraction on the same area before lamella preparation. Figure 2(c) displays the micro-XRD patterns highlighting the phases obtained: 3D, quasi-2D with *n* = 2, and *n* = 1. The patterns show only a single orientation for each phase, indicating the absence of multiple crystalline domains. In particular, the 2D phase exhibits a preferred orientation with the inorganic slabs parallel to the substrate plane, while the 3D phase aligns its *c*-axis along the plane of the substrate. The interfaces between the layers are sharp and the overall morphology is uniform, confirming the effectiveness of the growth method in achieving controlled phase separation and high-quality heterostructures. However, the heterostructure is not limited to a purely vertical arrangement. The observed lateral extension of the 2D domains indicates partial in-plane phase separation, accompanied by a nonideal vertical stacking of the coexisting phases. This mixed dimensional organization suggests a degree of anisotropic growth, likely driven by local solvent concentration gradients and differential nucleation kinetics across the substrate. Although lateral heterojunctions in similar systems have been reported in the literature,^34–36^ the realization of vertically stacked heterostructures demonstrated in this work represents a novelty of significance.

Optical microscopy and microphotoluminescence (μPL) spectroscopy, shown in Figure 3, were obtained for a large-area crystal, observed under white light at 4.5 magnification (Figure 3a). A magnified view of the crystal edge (20×) reveals a distinct layered architecture, where regions with different optical contrasts suggest the presence of multiple phases. Three representative regions (labeled 1–3) were selected for detailed PL analysis. First, the sample was examined under UV illumination using optical microscopy, in order to identify areas with distinct emission properties and guide the selection of regions for spectral acquisition (Figure S3). Subsequently, confocal μPL measurements were conducted in reflection mode under 450 nm excitation. The sample was excited from both the side A and the side B by flipping the sample, in order to excite the outermost layers on each side. It should be noted that the excitation light only illuminates a thin surface layer of the crystal, less than 100 nm in thickness, due to the absorption coefficient well in excess of 10^4^ cm^–1^ at the excitation wavelength. As shown in Figure 3e, under side A excitation, the two spectral features observed at 520 nm and at 580 nm are identified as the emission from the 2D and quasi 2D phases respectively (regions 1 and 2). As the 2D and quasi-2D layers are spatially offset relative to the underlying 3D perovskite phase, this spatial mismatch allows selective excitation of different regions, resulting in distinct emission signatures depending on the excitation spot. The near-infrared emission observed in region 3 corresponds instead to the 3D phase. Conversely, on the side B, only emission corresponding to the 3D phase at 800 nm is detected, indicating the predominance of this phase on the opposite side and confirming the vertical stacking of the heterojunction, since the excitation and collection paths do not access the quasi-2D domains located on side A.

**Figure 3:**
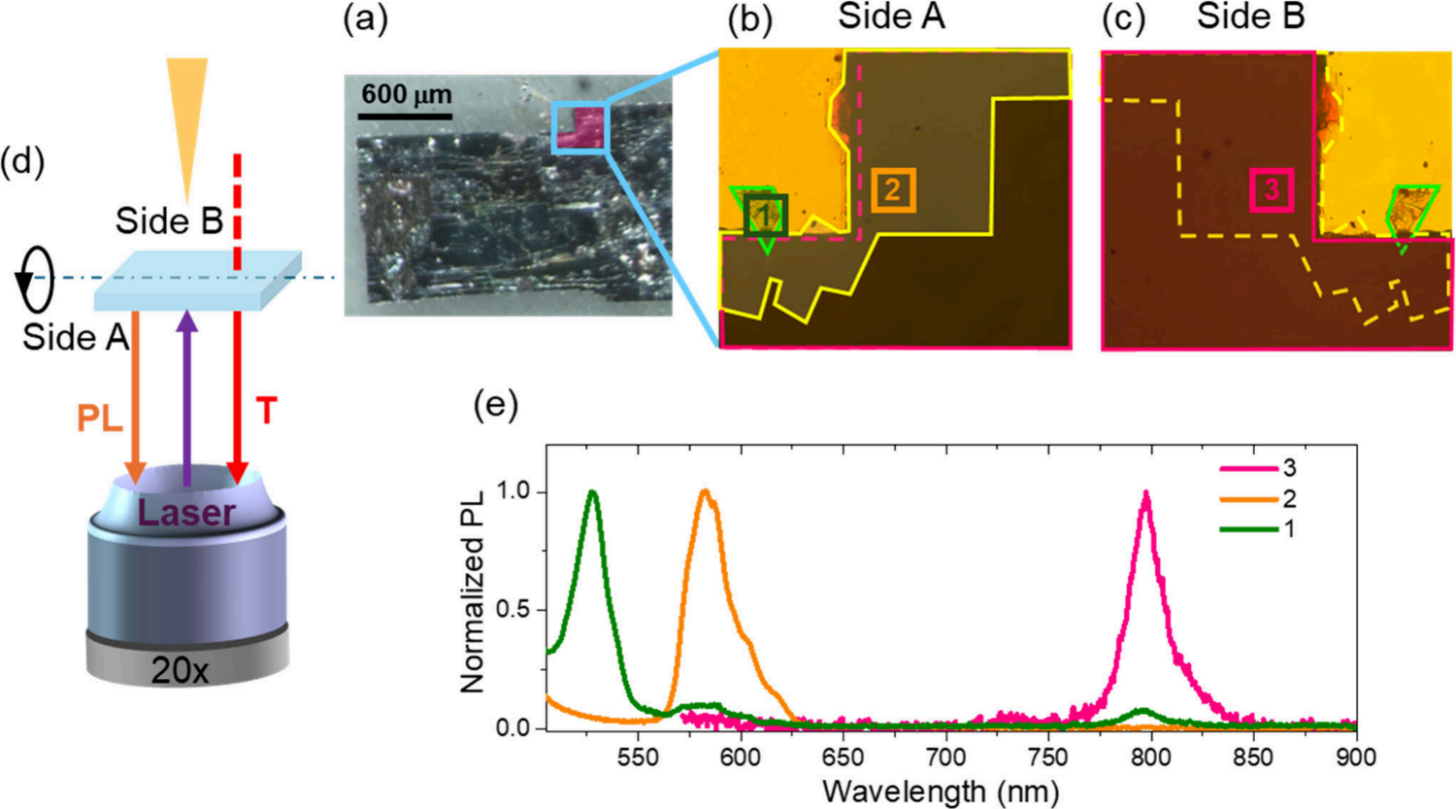
(a–c) Optical microscopy images of the sample. Panel (a) shows the full structure under white light in reflection geometry, with the region selected for magnification highlighted in magenta. Panels (b) and (c) display magnified views, in transmission geometry, of the region of interest from side A and the opposite face (side B) of the sample, respectively. In panel (b), the contours of 2D, quasi-2D and 3D crystals have been traced with green, yellow and magenta lines, respectively. Dashed lines indicate the underlying crystals, while solid lines indicate the overlying one. In panel (c), the sample is flipped to side B, placing the 3D crystal on top. The underlying 2D crystal, indicated by a yellow dashed line, is not visible in the optical image. The corresponding color PL maps, from which the crystal contours were extracted, are provided in the Supporting Information (Figure S3). (d) Schematic representation of the measurement configuration, showing the position of the objective lens and the excitation sources (white light and laser) relative to the sample surfaces. (e) Micro-PL spectra acquired with confocal microscopy in backscattering configuration at the positions labeled 1–3 in panels (b) and (c). Position 3 corresponds to the same area as position 2, but viewed from side B.

These observations support the formation of a controlled heterostructure, in which the sequential crystallization and spatial separation of different perovskite phases are achieved during the growth process. The ability to isolate and identify these regions highlights the tunability of the method and the potential to engineer complex architectures with tailored optoelectronic properties.

The crystallization mechanism is strongly influenced by the slow heating rate, which enables a progressive phase separation already at the solution stage, prior to nucleation. As the temperature rises, the retrograde solubility behavior of the precursor solution drives rapid supersaturation, leading to the nucleation and growth of the 3D perovskite phase with well-defined faceting (see ). As the 3D crystals grow, methylammonium MA^+^ ions (MA)-based precursors are progressively depleted from the solution, eventually halting further 3D phase development due to the lack of sufficient MA^+^ species.

The above mechanisms (schematically summarized in Figure 1a) have been further validated by large-scale molecular dynamics simulations. To this aim, we adopted the MYP2 force-field^47,48^ that extends the widely used MYP0 ^49–54^ to the study of crystallization phenomena. Figure 4 depicts an illustration of an atomistic model (panel a) consisting of a liquid solution of PEA_2_MA_4_Pb_5_I_16_ (i.e., 2D with n = 5), dissolved in GBL at a concentration of 0.083 molar fraction, in contact with preformed MAPbI_3_ (top) and PEA_2_PbI_4_ (bottom) crystalline surfaces (see the SI).

**Figure 4:**
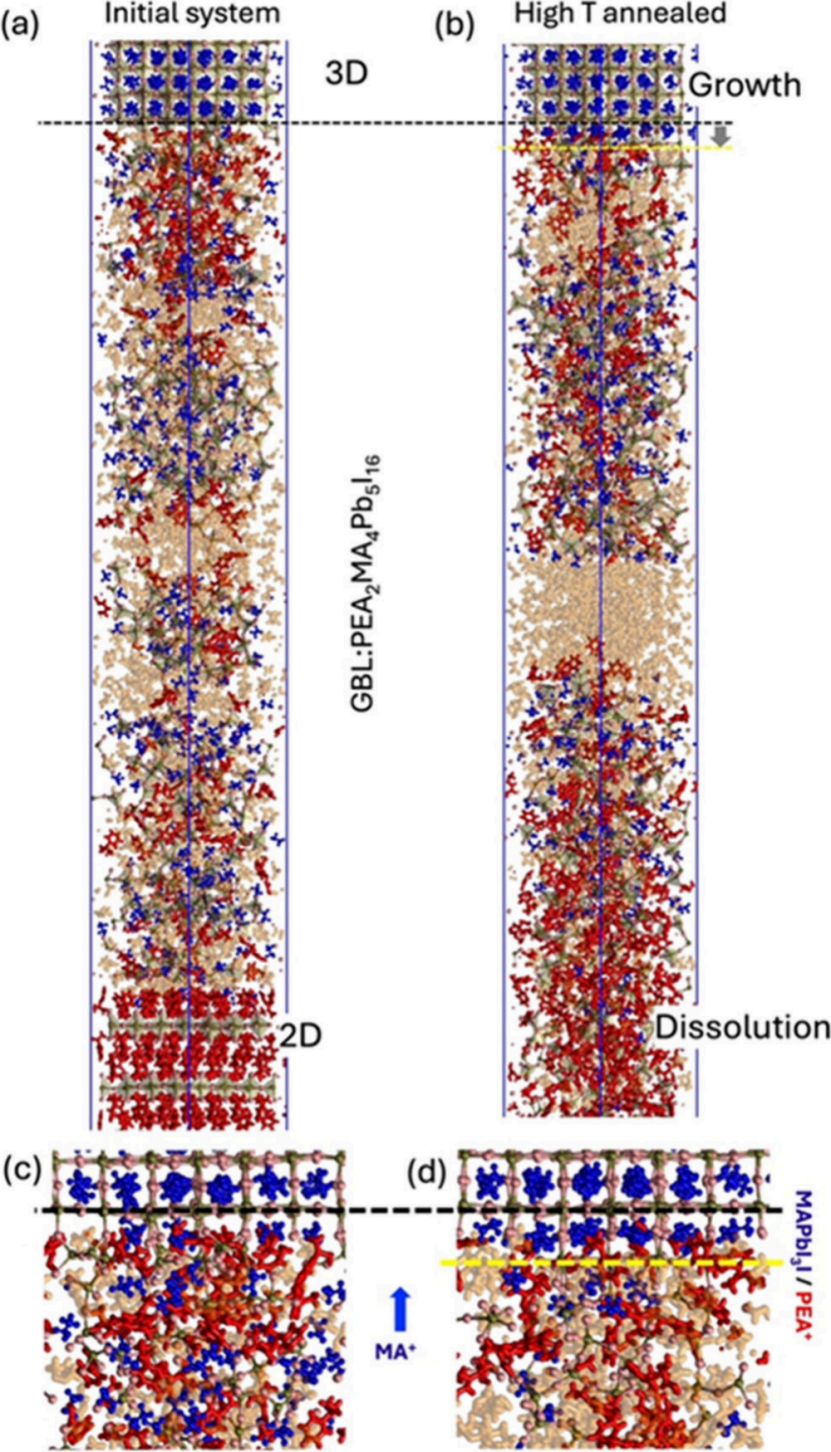
Panel (a): atomistic model of the initial system consisting of a liquid solution of PEA_2_MA_4_Pb_5_I_16_ (i.e., 2D perovskite with *n* = 5) dissolved in GBL solvent (0.083 molar fraction) in contact with 3D MAPbI_3_ (top) and 2D *n* = 1 PEA_2_MAPbI_4_ (bottom) crystals. Different colors represent MA^+^ (blue), PEA^+^ (red), Pb (green), I (pink), GBL (orange). Panel (b): the system after 60 ns annealing at high temperature shows the crystallization of the 3D MAPI with the advancement (gray arrow) of the surface (from black to yellow dashed lines); on the other side, due to the high temperature and direct solubility, the 2D perovskite crystal dissolves in GBL during annealing. Panels (c) and (d) report magnifications of the 3D/liquid interface in the initial and annealed system, respectively, showing the diffusion of MA^+^ molecules toward the 3D perovskite and the persistence of PEA^+^ molecules close to the crystalline surface.

A zoom of the MAPbI_3_ liquid interface is shown in panel c. During high-temperature annealing on the nanosecond time scale (panel b), we observe that the 2D perovskite crystal, in line with direct solubility, dissolves into GBL, while the 3D crystal, consistently with the reverse solubility, grows forming an almost complete crystalline layer (see Figure 4, panels b and d). This 3D crystal growth is enabled by the diffusion of MA^+^ molecules from the solution to the MAPbI_3_ surface. Interestingly, the 3D growth stops when the concentration of MA^+^ close to the crystal surface is low. At this stage, a predominance of PEA^+^ (red molecules) over MA^+^ (blue) is observed close to the 3D surface (see panels b and d). This simulation framework allows us to capture, at the molecular level, the interplay between precursor depletion and 3D phase growth, providing theoretical support for the experimental observations described above.

We hypothesize that the 3D nucleation occurs preferentially on the upper confinement quartz substrate, while a remaining solution underneath subsequently transforms into the 2D phase. This behavior is confirmed by microscopic observations, which clearly show the spatial distribution of the 3D crystals forming at the top and the 2D phases developing underneath (Figure S5).

During the heating stage, solvent evaporation occurs predominantly at the edges of the quartz substrates and this peripheral evaporation produces an increase in the concentration of solutes in the residual solution. This shift in the local equilibrium favors the nucleation of lower-dimensional 2D perovskite phases, typically *n* = 2 and *n* = 1 from the residual solution during the temperature decreasing. This crystallization process results in the spontaneous formation of a peripheral solid rim that partially seals the system. This self-limiting “sealing” effect restricts further solvent loss from the interior, thereby modifying the local crystallization environment without the need for external intervention. The 2D phase tends to grow along the edges of the 3D crystals, eventually overlapping and covering the 3D phase. For comparison, illustrates a case without the sealing effect: under these conditions, only two phases, 3D and *n* = 1, form, whereas with the sealing effect three distinct phases are observed, highlighting its crucial role in enabling the growth of the intermediate 2D phase with *n* = 2. The observation of 2D growth covering the 3D phase is supported by the MD simulations. As discussed above, after the 3D growth, we observe a fraction of PEA^+^ molecules remaining close to the 3D surface in the MA^+^ depleted region. This accumulation likely favors the crystallization of 2D phase (*n* = 1, 2) during the subsequent low temperature annealing. The growth of the 2D phase during the low temperature annealing is slow and not accessible in the nanosecond time scale. However, present results clearly show the tendency of MA to out diffuse from the GBL solvent (see Figure 4, panel c) to the 3D phase and the tendency of PEA molecules to remain close to the 3D phase (see panel d). These two factors are here identified as intrinsic molecular mechanisms of perovskite precursors in GBL contributing to the formation of the 3D/2D vertical heterostructures In conclusion, a one-pot synthesis approach is demonstrated for the controlled growth of a 3D/2D perovskite heterostructure. The proposed method demonstrates good reproducibility in terms of phase composition and crystal size. The 3D and 2D phases consistently form with the intended proportions, and the crystals always reach millimeter-scale dimensions. In spite of the simple and inexpensive solution process, temperature control is sufficient to produce clean interfaces between different crystalline phases, without the sophistication of epitaxial or high-vacuum techniques. The technique represents a bottom-up approach, where the crystal formation energy is temperature dependent and thus temperature control is sufficient to produce self-regulated growth of the desired perovskite phase. The produced heterostructures are stacked in the vertical direction with respect to the substrate, which is the desired configuration for all optoelectronic devices requiring thin perovskite films. However, at this stage, the technique does not allow precise control over the nucleation site, so crystals grow at random positions within the quartz slides. Improvements in deterministic control of crystal positioning could be achieved, for instance, by selective area functionalization of the substrate. Furthermore, preliminary investigations suggest that the method is readily extendable to other perovskite compositions, as long as their specific solubility behaviors are carefully considered. This strategy opens new perspectives for the integration of phase-engineered perovskite materials in optoelectronic devices.

## Supplementary Material





## Notes

Methods and experimental setups, reversible crystallization process driven by temperature and solvent dynamics, optical microscopy images of the heterojunction showing the overlap of three distinct phases, stepwise preparation of lamellae for STEM analysis and AFM characterization of the crystal surface including terrace profiling, two-photon photoluminescence mapping revealing the spatial distribution of the 2D and 3D phases along the *z*-axis, and computational details for crystallization phenomena (PDF)
crystallization process­(MP4)

## †

Ecole polytechnique fédérale de Lausanne, Institut des sciences et ingénierie chimiques, Lausanne, Switzerland
