# Supplementary material for: One-Pot Growth of 2D/3D Hybrid Perovskite Vertical Heterostructures
Source: ACS Mater Lett. 2025 Nov 6;7(12):3908–15. doi: 10.1021/acsmaterialslett.5c01109 (PMC12676453; doi:10.1021/acsmaterialslett.5c01109)
Supplement: Supplementary file 1 [file tz5c01109_si_001.pdf]

# Supporting Information

# One-Pot Growth of 2D/3D Hybrid Perovskite Vertical Heterostructures

*Selene Matta ,[1‡] Valeria Demontis,[1‡] Angelica Simbula,[1‡] Riccardo Pau,[1‡,†] Simone Argiolas,[2§] Alessandro Mattoni,[3∥] Silvia Liscia,[1‡] Ruirui Wu,[1‡] Nan Zhao,[1‡] Aditya Bhardwaj,[1‡] Francesco Mattana,[1‡] Emanuele Cadeddu,[1‡] Prajkta Liladhar Nehete,[1‡] Nicola Sestu,[1‡] Daniela Marongiu,[1‡],* Francesco Quochi,[1‡],* Michele Saba,[1‡] Andrea Mura,[1‡] and Giovanni Bongiovanni[1‡]*

*1‡ Dipartimento di Fisica, Università degli Studi di Cagliari, Cittadella Universitaria, 09042 Monserrato (CA), Italy*

*2§ Dipartimento di Fisica, Università degli Studi di Cagliari and CNR − Istituto Officina dei Materiali (IOM) Cagliari, Cittadella Universitaria, 09042 Monserrato (CA), Italy*

*3∥ CNR − Istituto Officina dei Materiali (IOM) Cagliari, Cittadella Universitaria, 09042 Monserrato (CA), Italy*

## Methods

*Materials:* Ammonium chloride ($NH_4Cl$, ≥99.5%), γ-Butyrolactone (GBL, 99+%), Lead Iodide (PbI2, 99.999 %), were purchased from Sigma Aldrich. Methylammonium Iodide (MAI, 99.99%), Phenethylammonium iodide (PEAI, 98.00%) were purchased from Greatcell Italy.

*Synthesis:* 2D perovskite $(PEA)_2(MA)_{n-1}Pb_nI_{3n+1}$ single crystals were synthesized using the space confined method. A 0.36 M precursors solution was prepared by dissolving PEAI:MAI:$PbI_2$:$NH_4Cl$ in stoichiometric ratios of 2:4:5:2 in 1ml of γ-butyrolactone (GBL). $NH_4Cl$ was used as an additive to modulate crystal nucleation and growth [1]. The resulting solution was stirred on a hotplate set at 70°C for 2h and subsequently filtered using a nylon filter with a pores size of 40 μm. Prior to crystal growth, two quartz slides of different sizes were cleaned by sequential ultra-sonication in soap, water, acetone, and isopropanol for 15 minutes each, followed by drying with $N_2$ flow. The substrates were then treated with an oxygen plasma asher for 3 minutes. Finally the larger slide was placed inside a temperature-controlled oven and covered with the other slide and then preheated at 100°C. A 10 μl droplet of the precursors solution was then deposited along the edge of the smaller substrate, allowing infiltration between them by capillarity. A weight of approximately (76 g) was placed on top of the system. The oven was programmed to follow a precise temperature cycle: the temperature was increased from 100°C to 140°C at a rate of 4°C/h. The temperature remained constant at 140°C for 10 hours. The cooling process followed a multi-step profile, combining several ramps at a fixed rate of 4 °C/h with intermediate stages at constant temperature. Specifically, the temperature was first reduced to 100 °C and held for 10 h. Then, the ramp was terminated at 100° in order to promote the preservation of the 3D perovskite phase alongside the quasi-two-dimensional phases by preventing resolubilization of the 3D structure. After the cooling step, the samples were placed under vacuum to complete drying and then the quartz substrates were carefully separated using a blade.

*In-situ investigation:* For in-situ characterization during the growth, we specifically designed an optically accessible growth reactor operating from ambient temperature up to 200 °C and customized to accurately control the growth parameters and to record optical images and videos using white-light optical microscopy. The oven consists of a brass central core and sample chamber, a manganin heating element and external thermal insulation made of Teflon. A bottom-mounted white LED provides sample illumination, which is observed through an optical system offering up to 10x magnification. The sample chamber is optically isolated from the external environment using quartz windows. The image is captured by a CCD camera and sent to a computer, which also controls the entire system.

Temperature regulation is achieved via a PID algorithm using two thermocouples: one located inside the sample chamber and another measuring room temperature outside the oven. This setup ensures temperature stability within ±1 °C across the entire operating range. The system can generate both heating and cooling ramps with customizable durations.

Precision stepper motors allow sample positioning with 10-micrometer resolution, enabling detailed observation of different regions for the detection of early crystal nucleation stages. The control software enables automatic image acquisition at any point during the thermal ramp, allowing accurate monitoring of sample behavior under controlled thermal conditions.

This approach allows us to investigate the crystallization kinetics in real time. A representative time-lapse extracted from these videos is shown in Figure 1c, while the full video is provided as supplementary material. This setup enables direct visualization of the sequential formation of the 3D and 2D perovskite phases during heating and cooling, providing valuable insight into the growth mechanism. The information obtained from optical inspection represents one element of the broader characterization framework required to understand the crystallization process in space-confined growth. These results need to be correlated with other techniques, such as XRD, STEM, and PL, performed after growth. Only the combination of all these methods provides a comprehensive picture of the heterostructure growth process.

*Characterization:* The optical microscope used for both bright-field and fluorescence analysis was a Nikon Eclipse TE-2000U. In white light, it operates in transmission mode, while fluorescence measurements are performed in reflection mode. Images were captured using a CS126CU Kiralux 12.3 MP Color CMOS Camera with USB 3.0 interface.
Confocal fluorescence imaging was carried out on the same Nikon Eclipse TE-2000U platform, equipped with a Nikon Digital Eclipse C1 laser scanning confocal system featuring excitation lasers at 405 nm, 488 nm, and 520 nm. Regions of interest for confocal imaging were initially identified in white-light transmission mode on the Nikon Eclipse TE-2000U. Fluorescence excitation for confocal acquisition used the 488 nm laser with all emission filters open to maximize sensitivity, particularly for samples with weak or overlapping emissions. Image acquisition and system control were managed via Nikon EZ-C1 software.

FIB lamellae were extracted with a ZEISS Crossbeam 550, equipped with a Ga ion source and working with in low-energy settings. TEM images were acquired with a JEOL TEM F200 Multipurpose working a 200 kV and equipped with Schottky field-emission gun (FEG) and a Gatan RIO-16 CMOS.

The perovskite diffraction patterns were collected using a powder diffractometer (D8 ADVANCE, Bruker) employing a Cu Ka radiation (40 kV/40mA) and a Goebel Mirror with UBC collimator 0.3 mm. The sample was placed on a UMC stage (motorized XYZ) and the patterns were recorded with the Detector LYNXEYE XE-T (1D continuous mode) with 0.03 deg/step and 0.5 s/step.

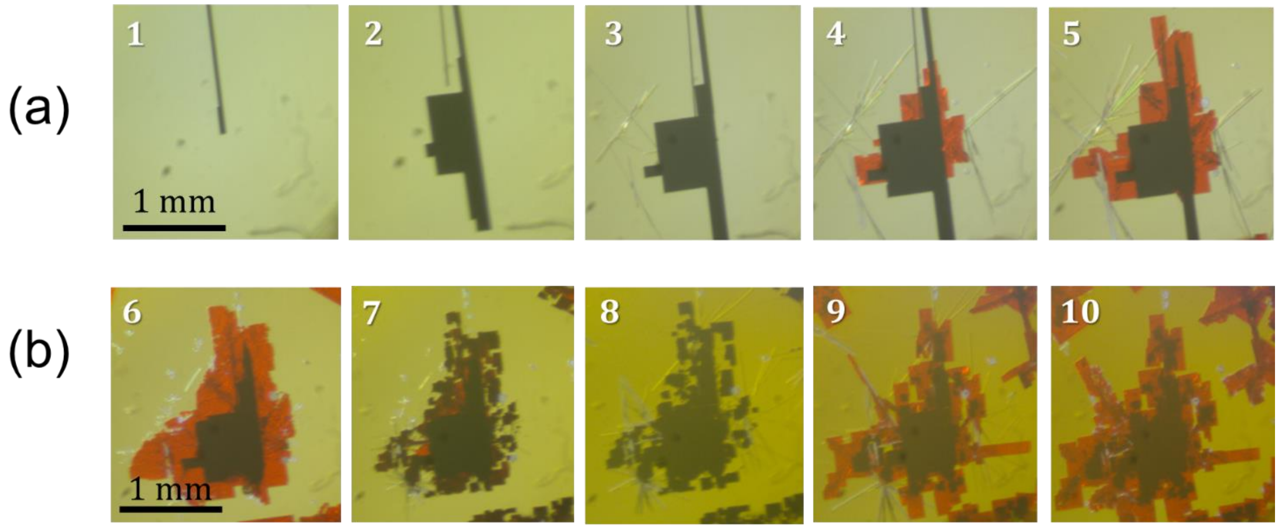


***Figure S1. Reversible crystallization process driven by temperature and solvent dynamics. Upon heating, inverse solubility induces nucleation and growth of faceted 3D perovskite crystals (1-3). During cooling, localized solvent evaporation promotes the formation of 2D phases that crystallizes from the homogeneous solution. Upon reheating, the 2D phase gradually dissolves, while the 3D phase simultaneously grows. the system can be cycled through these transformations repeatedly as long as sufficient solvent remains.***

When solvent evaporation from the substrates edges is minimized, the 2D phase with n = 1 no longer nucleates upon cooling. Additionally, under these conditions, the crystal growth exhibits reversible behavior: upon reheating, the quasi 2D phase redissolves and the homogeneous solution is restored. Only two well-defined and compositionally distinct phases, 3D and quasi 2D with n = 2 are observed throughout the process, with no formation of intermediate phases or n = 1. This confirms that solvent dynamics at the boundaries play a critical role in determining phase selectivity and reversibility.

To maintain solvent evaporation and allow the full progression of the crystallization sequence, including the emergence of the pure 2D third phase, it is necessary to manually disrupt this seal by removing the dried material from the substrate edges. This intervention re-establishes open boundary

conditions, facilitating additional solvent loss and shifting the crystallization equilibrium toward a three-phase coexistence regime (Fig. S4, S5).

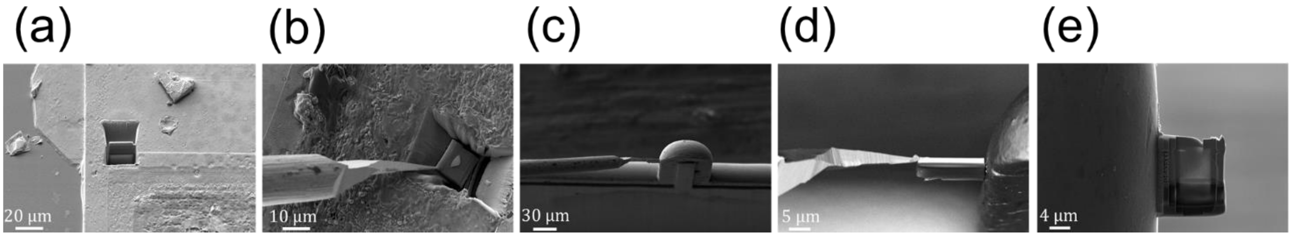


***Figure S2. SEM images of the main steps involved in lamella preparation: (a) FIB milling; (b) Nanomanipulator welding; (c) Lamella displacement; (d) Nanomanipulator detachment; (e) Lamella analysis with STEM.***

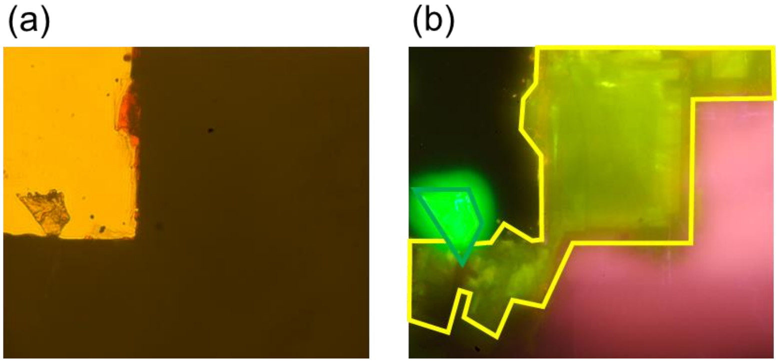


***Figure S3. Optical microscopy images of the sample under white light (panel a) and UV illumination (panel b). The UV image highlights the emission from the 3D perovskite phase, which appears pink due to the removal of the IR filter from the camera. These images were used to extract the crystal contours that are traced in the main text: the overlying 2D and quasi-2D crystals were outlined based on the UV emission, while the position of the underlying 3D crystal was inferred from the white-light image. The resulting contours were then used in the main text to illustrate the relative positions of the different phases.***

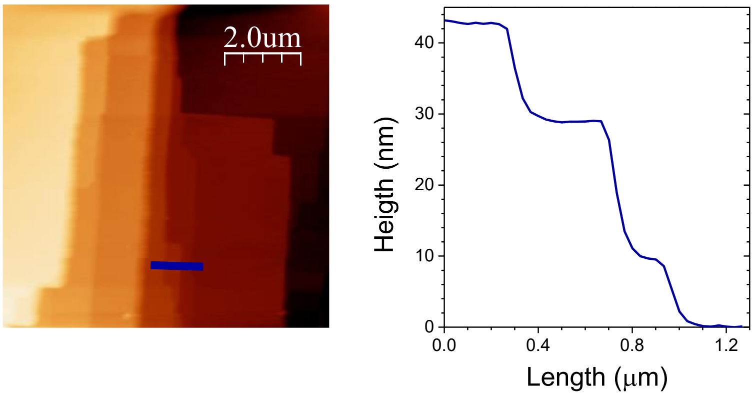


***Figure S4. AFM image of the 3D crystal surface, and corresponding profile of the terraces, acquired with a Horiba AINST-NT AFM, in non-contact mode.***

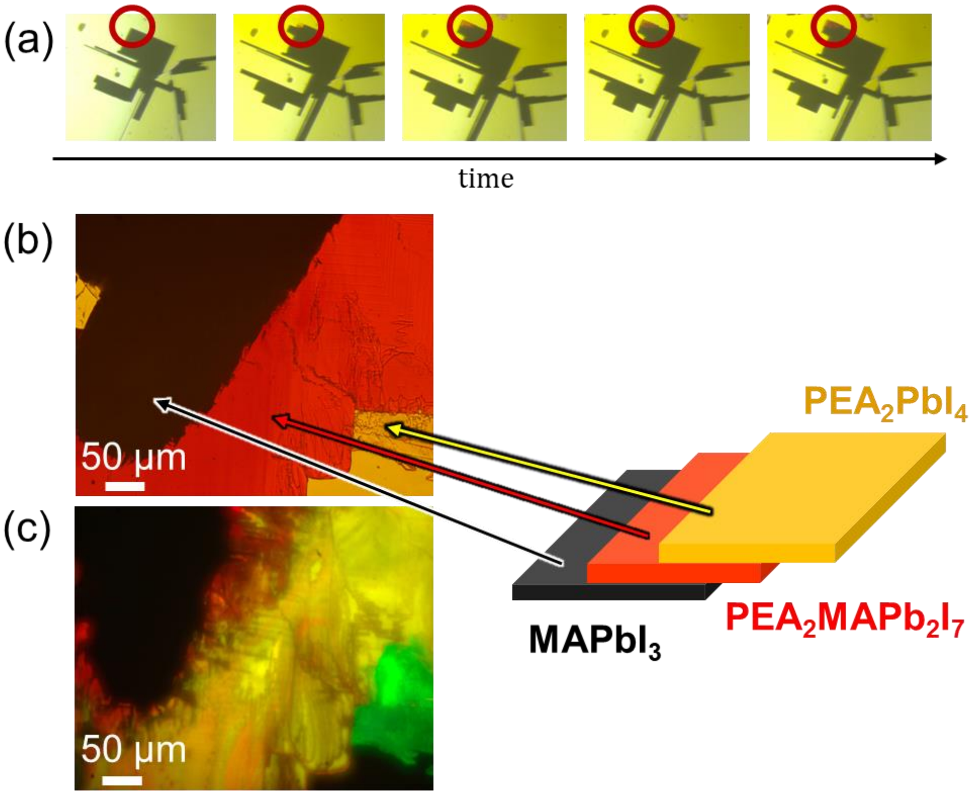


***Figure S5. Panel (a): optical microscopy time-lapse showing the growth of the quasi-2D phase originating at the edge of the 3D crystal. Bottom panel: optical microscope of the final triple heterojunction image under white light (b) and under UV light (c) accompanied by a schematic diagram illustrating the overlap of the three distinct phases in the layered structure.***

Figure S6 illustrates the crucial role of the self-limiting sealing effect in the 3D/2D heterostructure. The data show that, without the sealing effect, only two phases (3D and n = 1) form, whereas the presence of the peripheral solid rim enables the growth of the intermediate n = 2 quasi-2D phase, leading to the formation of three distinct phases within the heterostructure.

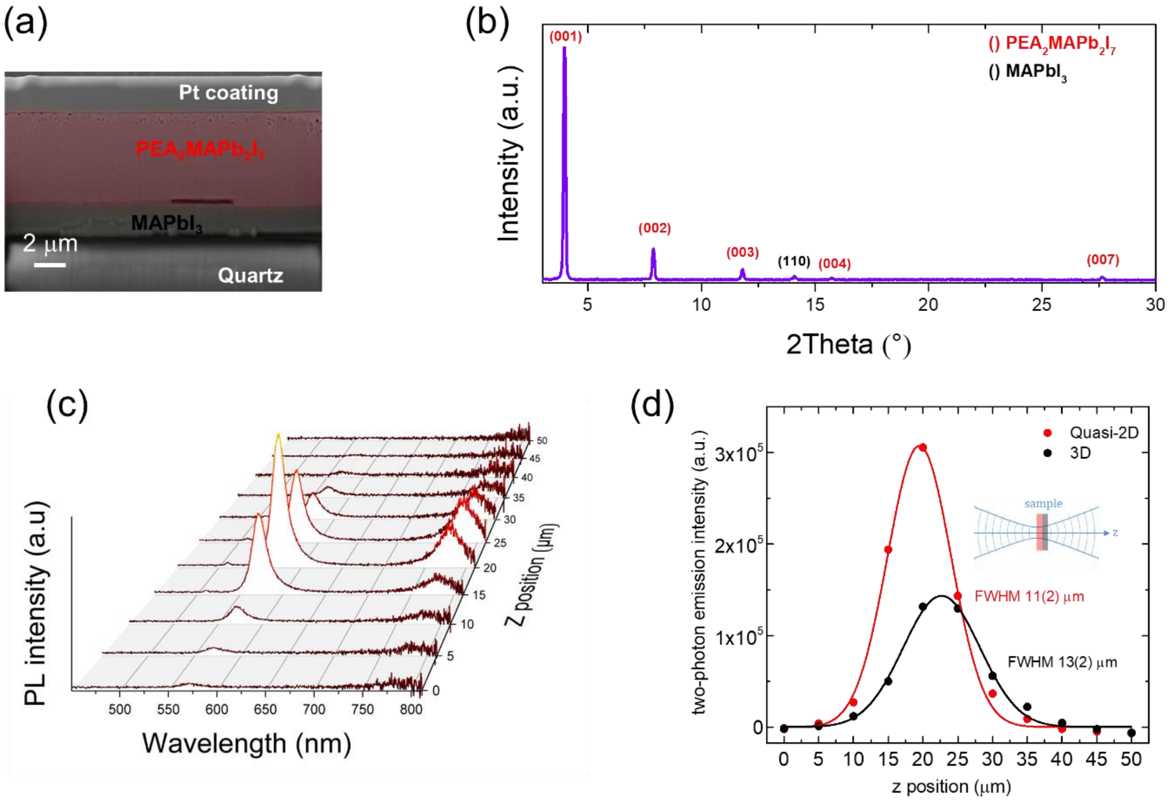


***Figure S6. (a) Cross-sectional SEM image of the 3D/2D heterostructure and (b) corresponding micro XRD pattern recorded before lamellae preparation showing the quasi 2D phase and the 3D phase. (c) Two-photon emission spectra recorded at different positions along the z-axis. The spectra reveal a depth-resolved optical response within the crystal heterostructure, clearly distinguishing two emission layers: one peaking at 580 nm, attributed to the quasi-2D phase, and another centered at 780 nm, corresponding to the 3D phase. (d) PL intensity integrated over the two emission peaks plotted as a function of sample position along the z-axis. Experimental data points (dots) are fitted with Gaussian curves (solid lines). The full widths at half maximum (FWHM) of the Gaussian fits are consistent with the laser confocal parameter, indicating the effective resolving power of the experiment. The separation between the two Gaussian peaks demonstrates the vertical stacking of the 2D and 3D phases within the heterostructure.***

Micro-photoluminescence (PL) measurements under two-photon excitation were performed using 100 fs pulses at a central wavelength of 1020 nm, emitted by an 80 MHz Ti:sapphire oscillator (Coherent Chameleon Ultra I). The laser pulses were focused onto the sample to a 1.8 μm spot with a 13 μm confocal parameter using a 40x microscope objective (Nikon Plan Fluor ELWD Series ). The sample was mounted on a longitudinal translation stage with micrometric resolution, enabling Z-scan-like measurements. The micro-PL signal was collected in reflection geometry and redirected via a dichroic mirror to a 150 gr/mm single-grating spectrometer (Princeton Instruments Acton SpectraPro 2300i, 30 cm focal length), equipped with a thermoelectrically cooled Si CCD detector (Andor Technologies Newton EM).

**Theoretical methods**

The simulated liquid mixture, $PEA_2MA_4Pb_5I_{16}$:GBL, contains 64 formula units of 2D perovskite with n=5. Each $PEA_2MA_4Pb_5I_{16}$ consists of 95 atoms and is formed from 11 precursor molecules: 2PEAI, 4MAI, $5PbI_2$. There are also 704 GBL molecules, each of which contains 12 atoms. The concentration of this $PEA_2MA_4Pb_5I_{16}$:GBL mixture corresponds to 0.083 molar fraction.

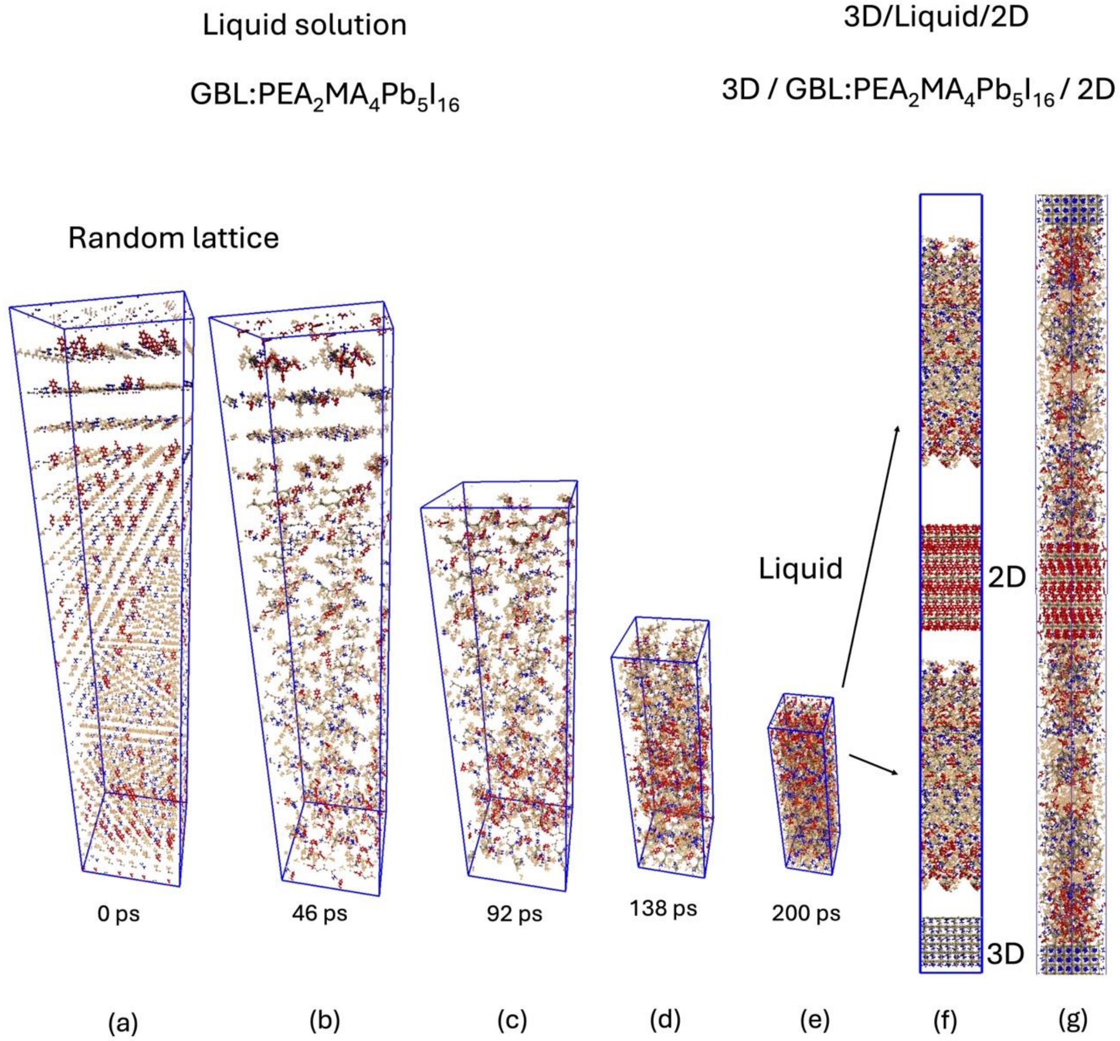


***Figure S7. (a-e) Preparation of the liquid solution from the random lattice; (f) preparation of the 3D/liquid/2D system; (g) initial 3D/liquid/2D system.***

The initial configuration was obtained by randomly distributing the precursors at the sites of a periodic grid: 128 PEAI, 256 MAI, 320 $PbI_2$ and 704 GBL molecules (see Figure S6, panel a). The periodic grid consists of 1408 sites, obtained by replicating a unit cell of size 1nm x1nm x 1.8 nm eight times in the x,y directions and twenty-two times in the z direction (8x8x22).The initial system was annealed for 200 ps in the NPT ensemble at 300 K (Fig. S6, panels a-e). During this time, the molecules moved closer together, the system density increased, and the initial order was lost, resulting in an equilibrated

liquid mixture. This mixture was then combined with 2D and 3D perovskite crystalline slabs (see Fig. S6, panels f,g). The 2D slab ($PEA_2PbI_4$, n = 1) was obtained by replicating the unit cell taken from Billing et al. [2] four times in the x-direction, four times in the y-direction and three times in the z-direction (4x4x3). The 3D slab consists of 4x4x3 tetragonal unit cells (the MYP2-optimized ones). Periodic boundary conditions were applied in all directions. By concatenating in periodic conditions the 3D system, the liquid solution, the 2D and again the liquid solution, we obtained the 3D/GBL/2D system containing 38,880 atoms (see Fig. S6, panel f) that was then annealed (see panel g).

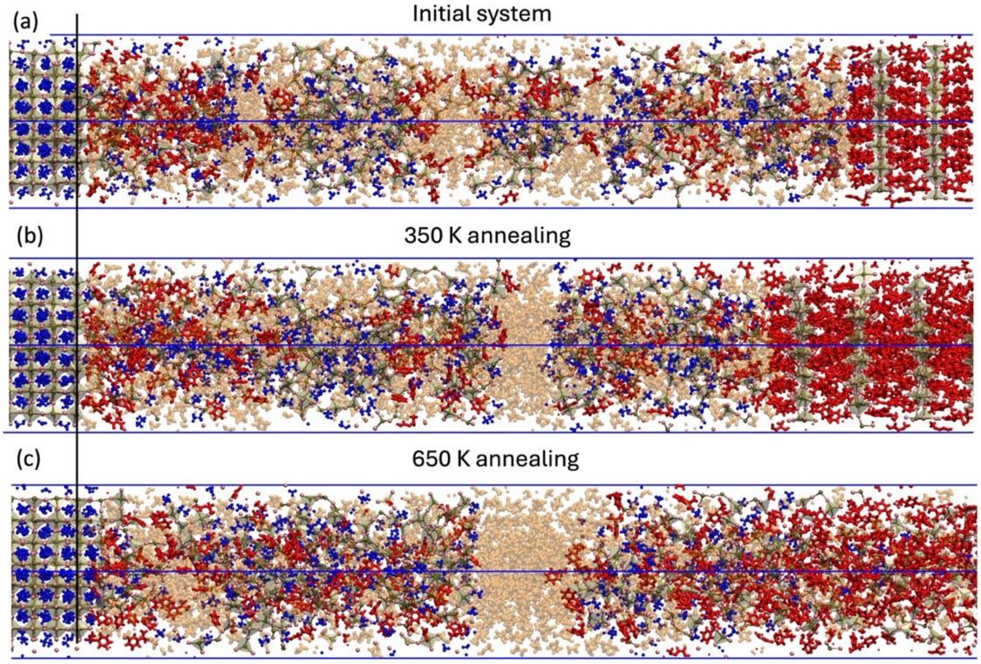


***Figure S8. Comparison between the initial (a) and annealed 3D/2D/liquid systems at 350 K (b) and 650 K (c); at 350 K temperature the 2D phase is preserved, while it is not observed a sizeable growth. At 650 K the 3D grows while the 2D dissolves into GBL.***

The interatomic forces were obtained by combining the MYP2 model for MAPI[3] with the GAFF[4] force field for PEA and GBL. We used the MYP2-derived Lennard-Jones parameters of Pb – Pb (e=0.0125 kcal/mol and s=4.05998 Å) and I – I (e=0.2099 kcal/mol and s=4.43090 Å) combining them to the standard GAFF by using the Berthelot-Lorentz mixing rules to obtain the missing interactions. Furthermore, the atomic partial charges of the GBL atoms obtained from AM1-BCC had to be decreased by a factor of two to observe the crystallization of MAPI in GBL during molecular dynamics simulations. The input files of the simulation are available as supporting material. Further optimization of the model parameters is beyond the scope of this work and will be the object of future investigation. To study crystallization mechanisms in solution under space confined conditions, the atomistic systems were annealed in NVT ensemble at temperatures between 350 and 650 K for at least 60 ns.

Crystallization occurs at the 3D surface and its velocity increased with temperature, with complete MAPI monolayer crystallization observed at 650 K and simultaneous 2D perovskite dissolution (see main text and Fig. S7, panel c). Interestingly, at 350 K (see Fig. S7, panel b), the 2D crystalline slab was preserved, while 3D growth did not occur.

**References**


[1] Oku, T., Ohishia, Y., Ueokaa, N. Highly (100)-oriented $CH_3NH_3PbI_3(Cl)$ perovskite solar cells prepared with $NH_4Cl$ using an air blow method. RSC Adv., **2018**, *8*, 10389–10395 DOI https://doi.org/10.1039/C7RA13582C.

[2] Billing, D. G.; Lemmerer, A. Synthesis and Crystal Structures of Inorganic–Organic Hybrids Incorporating an Aromatic Amine with a Chiral Functional Group. CrystEngComm **2006**, *8*, 686–695. https://doi.org/10.1039/B606987H.

[3] Mattoni, A.; Argiolas, S.; Cozzolino, G.; Dell'Angelo, D.; Filippetti, A.; Caddeo, C. Many-Body MYP2 Force-Field: Toward the Crystal Growth Modeling of Hybrid Perovskites. J. Chem. Theory Comput. **2024**, *20*, 6781–6789. https://doi.org/10.1021/acs.jctc.4c00391.

[4] Wang, J.; Wolf, R. M.; Caldwell, J. W.; Kollman, P. A.; Case, D. A. Development and Testing of a General Amber Force Field. Journal of Computational Chemistry **2004**, *25*, 1157–1174. https://doi.org/10.1002/jcc.20035.
